# Annotations

**Published:** 1890-02-08

**Authors:** 


### Annotations.

**Unpardonable.**

We are sorry that our excellent contemporary, the *Echo*, should have published a letter signed "Hugh Woods, M.D.," and entitled "Hospital Wastefulness." Our sorrow is due to the fact that the letter in question, one of very many which have emanated from the same pen of late, contains a series of charges against the employés of the metropolitan hospitals which we know to be unfounded and unjustifiable so far as the employés of institutions worthy of the name of hospital are concerned. Our regret that such a letter should have been published in any respectable newspaper is increased by the circumstance that the writer distinctly states that he declines to give his authority for the statements in question. We are glad to see that Mr. Leadbeater, M.R.C.S., writes to the *Echo*, calling upon "Hugh Woods, M.D.," to define and substantiate the charges he brings, on the ground that, if he thought it worth while to write the letter at all, he should have stated names and dates, and the institutions to which his remarks refer. If he can do this, his allegations may be worthy of consideration, but otherwise, as Mr. Leadbeater justly declares, "Very few men who have an M.D. degree which is worth having would have written such a letter." In emphasizing and reiterating this challenge, we are glad to note that the editor of the *Echo* states his agreement with Mr. Leadbeater, and adds that the writer should either have been prepared to substantiate the charges or to have remained silent. We beg to call upon Mr. Hugh Woods, M.D., to substantiate these charges or to withdraw them forthwith.

**Important to Charities.**

The case of Bazeley *v.* the President and Governors of the Royal National Hospital for Consumption, Ventnor, raised a point of great importance to charities. A certain Mrs. Hamilton, the widow of the Rev. William Frederick Hamilton, M.A., made her will in her own handwriting on December 10th, 1872, which contained the following bequest:

> I give to the said trustees and executors upon trust the Government stocks or securities that may be remaining, and the money at my bankers belonging to me at the time of my death, to enable them to pay off all my just debts, &c., and the residue of the said remaining Government stocks or securities I direct shall be given to the trustees for the time being of the National Hospital for Consumption and Diseases of the Chest, Ventnor, Undercliffe, Isle of Wight, upon trust to stand possessed of the same, and also of whatever money of mine may then be remaining at my bankers should there be a surplus upon the trust following—namely, to set apart sufficient money for the payment of a stipend of £150 per annum to a respectable clergyman of the Church of England, holding sound Evangelical doctrine, and a good reader for the purpose of reading or preaching to patients in the hospital, or in the hospital chapel every week one or at least portions of one of my beloved husband's sermons.

And the testatrix directed her residuary estate to be applied for the purpose of receiving gratuitously into the hospital poor persons of respectable character. The testatrix died in 1889. She did not possess at the date of her will, or at the date of her death, any Government stock or securities of the United Kingdom, but only certain New South Wales and Victoria Government bonds, and some Indian railways stock which was guaranteed by the Indian Government. At the date of the will the only Government securities of any kind which the testatrix was known to possess were certain Victorian bonds. The executors did not oppose the handing over of the Colonial Government and Indian securities but, as the will was vague, they went to the Court for authority. In the result, first Justice North, and subsequently on appeal Lord Justice Cotton and Lord Justice Lindley, Lord Justice Lopes dissenting, decided that Colonial securities, or Indian railway stock, guaranteed by the Indian Government, cannot be held to be included in the term "Government stock or securities." We believe this decision to be wholly wrong both in principle and fact. In fact, because the words "Government stock or securities" in the money market sense do most certainly include "Colonial securities and stock guaranteed by the Indian Government"; and the classification of securities in the schedule of the Life Insurance Companies' and other Acts follows this practice of the markets. The decision is further contrary to principle, as Lord Justice Lopes rightly maintained, seeing that it was the clear intention of the testatrix in this particular case to hand over the Colonial and Indian railway stocks in her possession for the benefit of the Consumption Hospital at Ventnor. We hope, therefore, that the committee of the hospital will carry the case to the House of Lords, when we have little doubt that the extraordinary misapprehension under which Mr. Justice North and Lords Justices Cotton and Lindley evidently laboured will be removed, and a decision will be given more in accordance with sound principle and admitted practice.

**Pauper Lunatics in Licensed Houses.**

The *Echo* has a letter on this subject, and although we cannot accept the inference that pauper lunatics are detained in these asylums after recovery, either for the sake of their labour or for money received for maintenance, we are strongly of opinion that the system of allowing pauper patients to be detained in private asylums is indefensible, even if we look no further than the additional expenses which the system entails. The number of pauper patients in the metropolitan asylums may not be accurately known to outsiders; but it is probably not far short of one thousand. Now the charge to the guardians for each of these patients is something like nineteen shillings a week, while the charge in county asylums is only nine. If "Awake" will reckon up what this means for one year, and will multiply the sum he gets by the number of years it has been going on, he will be surprised at the amount which the Middlesex ratepayers have been compelled to pay. If "Awake" will refer to the 16 and 17 Vic., c. 96., s. XIX., he will find that the superintendent of the private asylum is bound to give notice of the recovery of any pauper patients to the Guardians; and the 8 and 9 Vic., c. 100., s. LXXIV., provides that the Guardians may order the removal of any pauper from the private asylum, unless the medical officer shall certify that the patient is dangerous and unfit to be at large. But even in this case the Commissioners have power to order the patient's discharge. Such are the Acts, and it would probably not be difficult to put them in force in the case under notice.

**The Last of the Influenza.**

The epidemic is almost moribund. We are, most of us, convalescent, and find the period of convalescence a long and dreary time. It is well for us if physical weakness and languor are the worst we have to fear, if we dare recover at our leisure without being harassed by the thought that idlenes means hunger to ourselves and those we love. Very few of the fatal cases that have occurred have been due to influenza alone; as a rule they have resulted from some rash or premature exposure. It is all very well for the doctor to say, "Don't think of going out for a week yet, and then only if the weather is fine." The patient knows what another week's idleness means, and when it is a choice between dying of hunger and dying of bronchitis, he is apt to chance the bronchitis. Then if his food is secure he needs something more than the daily bread he required in health. The capricious appetite wants stimulating, and even that strong advocate of temperance, Dr. Symes Thompson, declares that stimulants are often desirable. One of the saddest things about illness is that extra demands are made on the purse just when it is most empty. Here, then, is a legitimate field for charity. It is not far to seek. There are few of us who cannot find near our own door some case where a friendly gift of some little dainty would not be a welcome thing. Our influenza will not have been all evil if, in leaving, it gives occasioning for that strengthening of the bonds of mutual help which forms the firmest foundation of human brotherhood.

